# Characterization of a COTS-Based RF Receiver for Cubesat Applications †

**DOI:** 10.3390/s20030776

**Published:** 2020-01-31

**Authors:** Antonio Lovascio, Antonella D’Orazio, Vito Centonze

**Affiliations:** 1 Dipartimento di Ingegneria Elettrica e dell’Informazione, Politecnico di Bari, 4, E. Orabona St., 70125 Bari, Italy; antonella.dorazio@poliba.it; 2 Space Instrument & Avionic Division, Sitael S.p.A., 21, San Sabino St., 70042 Mola di Bari, Italy

**Keywords:** COTS, cubesat, radiation test, receiver, RF, satellite

## Abstract

This paper reports the experimental results of a test campaign performed on the radio-frequency (RF) receiver prototype operating at a 2025–2110 MHz frequency range, designed and fabricated for CubeSat applications. The prototype has been tested through a board-level test approach for the verification of the functional requirements and a component-level one for specific characterization measures. The tests have shown the following results: a −115–−70 dBm sensitivity range, 390 MHz intermediate frequency, a 0 dBm output power level with ±1 dB error, a 2.34 dB noise figure, and a 4.86 W power absorption. Such results have been largely achieved implementing an automatic gain control system by cascading two Commercial Off-The-Shelf (COTS) amplifiers. Moreover, an innovative technique based on RF test points has been successfully experimented and validated to measure the S-parameters of a custom low-pass filter integrated on the receiver, showing the possibility of even characterizing the single COTS components exposed to radiation through a unique board-level test setup. The technique may have a great impact on the cost reduction of electronic boards for space applications, since it would avoid using expensive evaluation boards for each COTS component that needs a radiation test.

## 1. Introduction

Recently, the research on the framework of the aerospace applications has been devoted to CubeSats because they are small and compact, characterized by low launch costs, and suitable to create satellite constellations that can improve the terrestrial network infrastructure, providing better global coverage.

The CubeSats are typically designed using Commercial Off-The-Shelf (COTS) components. The use of COTS components has the great advantage of widely reducing the costs of the satellite subsystems, since additional screening and reliability tests/inspections are not mandatory. Therefore, in recent years, they have generated a great deal of interest from the industry of small satellites, especially for Low-Earth Orbits (LEOs) [1,2].

The LEOs are the best environment for the allocation of hardware based on COTS components. The satellites find a less severe environment in such orbits, within which the components, even if not space-qualified, could “survive” the strong temperature ranges and the ionizing radiations.

The use of COTS components for space missions is well documented in the literature. The COTS components are used to make both the power electronic [3,4,5,6] and digital [7,8,9] boards. The term “COTS” is also related to the test facilities [10,11] and electrical ground support equipment [1,12]. Finally, the COTS-based design has been even adopted for RF systems [13,14,15,16,17,18,19,20]. They include navigation systems [13,14], transmission data systems for deep-space and remote-sensing applications [15,16], and modules for communication with the earth [2,17,18,19,20].

The main problem in the use of COTS components in space applications is the increased risk of failure of the satellite subsystems that implement them. Their suitability for use in space must always be verified by looking for reliability information in the literature, mostly related to radiation tolerance. Some selection criteria of COTS components, even standardized, have been developed in recent years to help designers to make a reasonable choice of component considering the orbit where the satellite will be placed.

The main issue of an approach based on selection criteria is related to the limited available information associated with the radiation tolerance of the COTS components. Since radiation is the main cause of failure in the space, the components are often tested for their tolerance to radiation under appropriate conditions, and the corresponding test reports are filed in databases which are not always freely available. Moreover, most of the components for which a radiation test report is available are components for low-frequency and power applications. Few Radio-Frequency (RF) components are available in such databases. For this reason, the design of COTS-based RF front-ends that have high-performance and reliability at the same time is particularly difficult.

In the literature, there are few papers that report RF front-ends implemented with COTS integrated circuits [21,22,23], for which reliability information and radiation test reports are more easily available. However, such an approach solves the reliability issue, but does not always allow the creation of applications with very high performance, since the integrated circuits are designed to be general-purpose for terrestrial applications.

The intent of this work is to find an efficient solution to deal with this issue, considering as a case study the design of an RF receiver operating at a 2025–2110 MHz frequency range, conceived for CubeSat applications. The starting idea was to design a completely custom device with a high performance and high reliability using only COTS components, even without having information regarding their behavior under radiation. Such design requirement has been addressed from a purely analytical point of view in our original work, presented at the Metrology for Aerospace 2019 conference [17]. This article is an extension of that work, where the same design requirement has been addressed from an experimental point of view, i.e., through the adoption of an innovative technique based on the use of RF test points. The adoption of this technique has driven the design of the electrical schematic and the layout, up to the fabrication of a functional prototype. The prototype has been tested and the experimental results are reported in this paper. Moreover, a detailed characterization of a component performed by means of the RF test points is presented, proving definitely that the use of RF test points allows the individual testing of the component, insulating it from the rest of the circuit, which is extremely attractive if radiation tests are executed on that component along with the whole board. In other words, by performing a unique radiation test on the board, information regarding the radiation behavior of each single component can be obtained, saving costs and avoiding the use of expensive evaluation boards.

## 2. The Method Extension

The RF receiver was designed to operate with a 2025–2110 MHz input frequency range to be compliant with the European Cooperation for Space Standardization. A design flow, typically adopted in the aerospace context, was followed. Starting from the mission analysis, a minimum set of system requirements was defined, which constituted the starting point of the design, especially in the choice of the most suitable architecture.

The superheterodyne architecture was adopted for the receiver design. The architecture foresaw a single conversion stage to minimize the number of the components. The RF incoming signal was downconverted to the Intermediate Frequency (IF) by a COTS RF mixer, amplified, filtered, and finally output through a coaxial connector.

With respect to the traditional architecture, some improvements were implemented to meet the noise figure and input dynamic range requirements. The noise performance has been improved by cascading two low-noise and high-gain COTS amplifiers before the preselector filter, which allowed a drastic decrease in the total noise figure. Likewise, two cascaded adjusted gain control COTS amplifiers were placed at the end of the receiver chain, which allowed to obtain a gain variation dynamic of 52 dB that, besides fulfilling the input dynamic range requirement (−115–−70 dBm), also added 7 dB of margin.

In conference paper [17], we focused on the modelling aspects of the system. The receiver model was made considering not only the nominal functional performance but also the performance in the worst-case. We pointed out that the “worst case” is not easily identifiable; therefore, it was implicitly assumed in the system requirements. For example, if the noise figure must be smaller than 3 dB by requirement, such requirements must be valid even in the presence of radiation and thermal changes. Another assumption was that the RF parameters drift from the nominal value because of frequencies, operating temperature, and total ionizing dose. The amount and the signs of such drift were not known a priori, because of the lack of reliability data.

Since the final target of the receiver is to amplify the incoming signal to a fixed output power of 0 dBm ± 1 dB (by requirement) and since the RF parameters suffer a drift, as previously assumed, we implemented a worst-case analysis based on the Monte Carlo analysis that allowed to estimate the total net gain and noise figure of the whole receiver, starting from the variation of the RF parameters of each individual component. Such an estimation was performed assuming a safety margin that considered both lack of reliability information and the lack of the specific tests on the evaluation boards, i.e., using only the data reported in the datasheets, even if they were less accurate. The safety margin was set at 10%, resulting from a reasonable hypothesis on the components’ behavior when studying their technology.

The system simulations showed that all requirements were satisfied. The most relevant result was in the output power level, which was equal to 0 dBm even in the presence of net gain variations that exceed the minimum required input dynamic range (45 dB). In other words, the Monte Carlo analysis has shown that the 7 dB of margin obtained by the AGC system can compensate any drift in the output signal power level.

The assumption that RF parameters only suffer a drift may be too conservative. Especially concerning radiation, we cannot exclude a priori the possibility that the ionizing radiation could cause permanent damage to the components. Therefore, the components’ behavior can be definitively predicted only by performing a component level radiation test. Such a target has been reached and presented in this manuscript. The adopted method is based on the experimental tests designed to be performed even in the final phases of the project. The innovative element is in using RF test points, simple COTS connectors, not only to guarantee the functional performance of the receiver, but also to enhance the reliability of a device intended to be used in very severe environments. The method extends and improves the analytic approach reported in the conference paper [17] since it allows to complete the system model so that the results’ accuracy is improved.

## 3. RF Receiver Prototype Description

**Figure 1:**
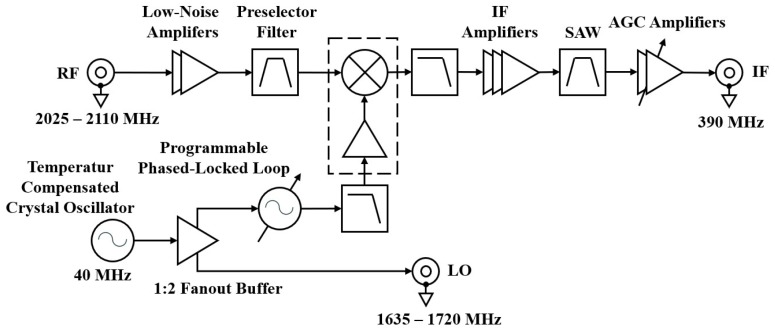
Radio frequency (RF) receiver block diagram.

**Figure 2:**
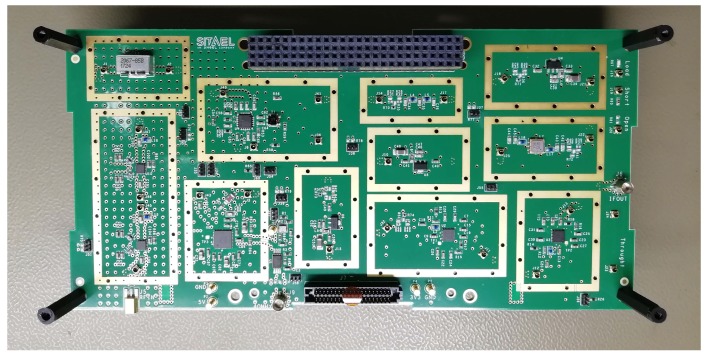
A photograph of the RF receiver prototype.

Figure 1 and Figure 2, respectively, show the block diagram of the RF receiver and a photograph of the assembled prototype.

The Printed Circuit Board (PCB) is made of eight layers, using a 0.254 mm RO4350B substrate as dielectric core for the top and bottom layers and RO4450B substrates for the prepreg inner layers. The stack-up is about 2 mm thick and has 192 mm × 95.9 mm size. One of the two board dimensions has been enlarged (with respect to the 1U CubeSat standard) to allow the placement of the RF test points, which normally are not placed in the flight model of the same system.

All RF paths have been routed on the top layer, while the bottom layer has been reserved for a few secondary components. Moreover, there are three inner ground planes, two planes for the power supply routing (one for the 5 V and the other one for the 3.3 V voltage), and a plane reserved for shielding issues.

In Figure 2, the PC104 and other secondary connectors are evident. Such connectors were the first components to be placed on the board, since they are a mechanical constraint. The schematic and layout of the components were designed by following the guidelines of their reference design, reported in the datasheets, trying to reproduce the placement of the lines, components, and via-holes. The lines that connect the two components have been routed following the logical connection of the high-level system, i.e., of the reception path. The technology of the coplanar microstrips has been chosen to design such lines. Their characteristic impedance has been optimized to 50 Ω because all selected components operate with a 50 Ω reference impedance. In this way, the matching impedance has been guaranteed. With the RO4350B substrate that has 3.48 dielectric constant, a 50.2 Ω characteristic impedance has been estimated, with a 0.52 mm microstrip width and a 0.28 mm gap between the microstrip and coplanar ground plane.

For each active element, a metallic shield has been introduced to reduce the electromagnetic emission and to avoid cross-talk and interference from nearby components. Being a prototype, commercial shields with a prefixed size have been used. They are composed of a frame and a removable cover. The frame is the part of the shield that is mounted on the PCB through via-holes and soldered on copper pads. The cover can be removed, if necessary, for debug operations and to rework the board. The shields are 5.08 mm height and are made of 0.38 mm thick tin-plated steel.

## 4. Test Method

The experimental characterization of the prototype has been done by adopting two main types of tests: component-level tests and board-level functional tests.

### 4.1. Component-Level Test Description

The component-level tests have allowed to verify that, for each component, the schematic and layout implementation have been correctly designed and no issues have occurred during the prototype’s fabrication and components’ mounting. The main purpose of this type of test is to measure the main functional parameters of the RF components, such as the power gain, the input and output return loss, the 1-dB compression point, and the noise figure. The execution of such a test has been made possible thanks to the adoption of the RF test points. They are typically used to monitor the power level along the signal path to control and improve the design of a system.

**Figure 3:**
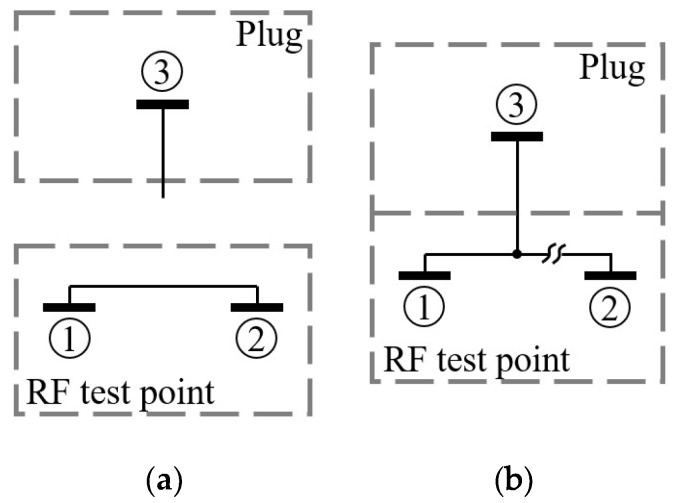
Operating scheme of the RF test point selected for the RF receiver prototype: (**a**) not-mated to plug; (**b**) mated to plug.

The RF test points selected for the receiver are surface-mount connectors that have 2.3 mm × 2.3 mm × 1.35 mm size and can operate from Direct Current (DC) to 11 GHz [24]. Figure 3 shows their basic operating principle.

When the plug is “not-mated” to the connector, the “1” and “2” pins are short-circuited. From the signal point of view, that short-circuit is a piece of metal (2.3 mm long and 0.35 mm width) that exhibits a small inductance, negligible at the receiver operative frequencies. The voltage standing wave ratio is about 1 at 2 GHz and the attenuation is smaller than 0.1 dB [24].

When the plug is “mated” to the connector, the “1” pin disconnects from the “2” pin and it connects to the “3” pin that is the positive of the plug. If the “1” pin is connected to the input or output of an RF component of the receiver, the “1” pin would allow access to the input or output of that component as if it were an evaluation board. The plug breaks the RF signal path on the board and routes it toward the plug itself, which can be a coaxial connector linked to a measurement instrument.

The basic operation of the RF test point has been exploited to form the input and output ports for each component of the receiver. Each port (regardless if it is an input or output) has been made by connecting the “1” pin of the test point to the input/output pin of the RF component. In this way, the component can be isolated from the rest of the circuit and can be tested individually. For the component-level tests of the active elements, some mechanical switches have been foreseen for debug purposes. The switches allow to power on the active components individually to avoid the influence of the other components under test. Moreover, the switches provide the possibility to shut down the whole board when passive elements are under test.

The RF test points are very effective to measure the S-parameters of the passive components (as the filters) and, as it is known, the S-parameter measurement requires an appropriate calibration kit that must be compatible with the mate-connector used to connect the network analyzer to the RF test points. To perform a more accurate network analyzer calibration, the calibration kit has been custom-designed on the same receiver board, rather than purchasing a commercial one. Such a choice has also been cost-effective because, as the calibration frequencies were smaller than 3 GHz, the custom calibration kit could have been designed using lumped components, especially for the 50 Ω load.

**Figure 4:**
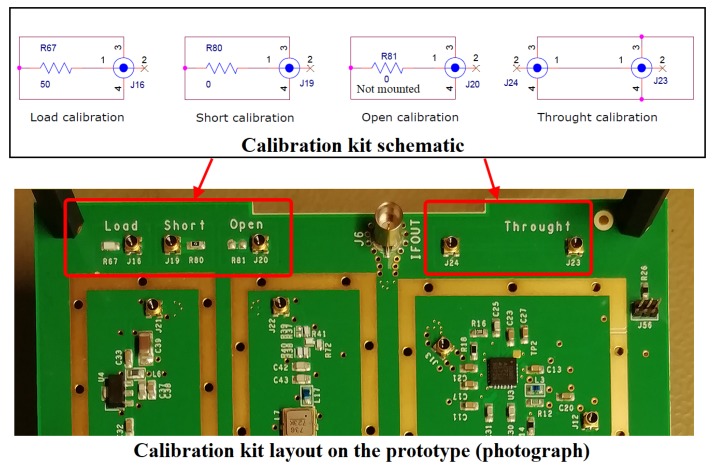
Custom calibration kit for the mate-connector of the RF test points. Top image shows the schematic and bottom image shows a photograph of the its implementation on the prototype board.

Figure 4 shows the calibration kit schematic and a photograph of its implementation on the prototype board.

The calibration system of the “load” was made using a 50 Ω resistor with very high accuracy, i.e., ±0.1%. The calibration system of the “short” was made using a 0 Ω resistor. The calibration system of the “open” was made foreseeing a simple footprint without any component mounted on the board. Finally, the calibration system of the “through” was made by designing a coplanar microstrip with a 50 Ω characteristic impedance. The length of such a microstrip has been assumed to be equal to the average electrical length of all components placed on the board.

The schematic of the calibration kit, illustrated in Figure 4, also shows that the return signal is not connected to the same receiver ground to prevent all possible eventual errors in calibration caused by the rest of the circuit. The calibration of the network analyzer was always performed with the receiver off.

Besides the aspects related to the calibration, the implementation of the RF test points required attention for the encumbrance and mounting aspects. Regarding the encumbrance aspect, to save space on the board, a 3D analysis has been performed to search for the minimum spacing to place two near to the RF test points (estimated as equal to 0.4 mm) in order to avoid the mate-connector touching the nearby RF test point, preventing the insertion. The 3D analysis has also helped to estimate the right height of the metallic shields (5 mm). Regarding the mounting aspect, since the RF test points have a small size and the mounting has been done by machine, the footprint design of the connectors has been customized in order to provide the correct guidelines for the operator who physically mounted the components, orienting it correctly. An error in the component orientation would have compromised the whole test approach.

### 4.2. Board-Level Test Description

The board-level tests have allowed to verify the functional performance of the whole receiver from the electrical point of view, i.e., to verify its compliance with the high-level requirements defined in [17]:

- Sensitivity range: −115–−70 dBm;
- Noise figure: <3 dB @ −115 dBm;
- Intermediate Frequency: 390 MHz;
- IF output power: 0 dBm ±1 dB;
- DC power absorption: <3.5 W.

The verification of these requirements needs a test setup that links the instrumentation to the main external interfaces of the receiver, i.e., the coaxial ports RF, IF, Local Oscillator (LO), and the access points for the power supply voltages. The board-level test requires that the whole board is switched on, so that all amplifiers allow the passage of the signal from the input (the RF) up to the output (the IF). Moreover, the power-on of the board allows to measure the DC power absorption.

## 5. Test Results

### 5.1. Measure of the S-Parameters of the IF Low-Pass Filter

In this subsection, an example of a component-level test exploiting the RF test points is shown. The S-parameters have been measured on a low-pass filter placed on the receiver IF section. The IF low-pass filter has been custom designed to reject the LO spur that couples on the IF, because of the low insulation of the mixer ports. The measure of the S-parameters has allowed to verify the filter performance and to validate the simulation model.

**Figure 5:**
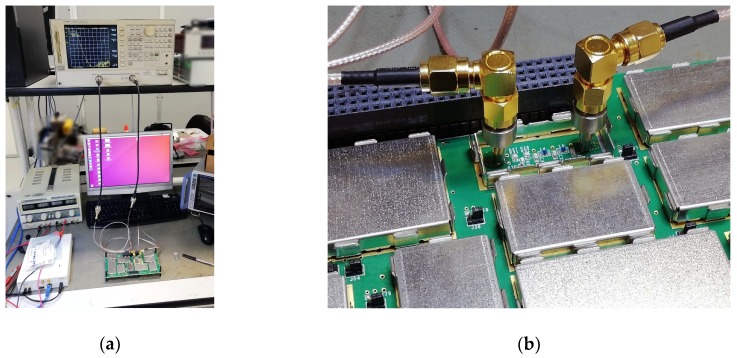
Photograph of the test setup implemented to test the custom IF section (390 MHz) low-pass filter: (**a**) The whole test setup; (**b**) A zoom on the mate of the instrument connectors to the filter RF test points.

Figure 5a shows a photograph of the test setup, which is constituted of a network analyzer [25] that has been connected to the filter through two mate-connectors. Figure 5b shows an enlarged photograph of the connection of the mate-connectors to the input and output RF test points foreseen for the filter.

The network analyzer has been calibrated using the custom calibration kit shown in Figure 4 within the 30 kHz–3 GHz frequency range, setting the test power level equal to −30 dBm.

**Figure 6:**
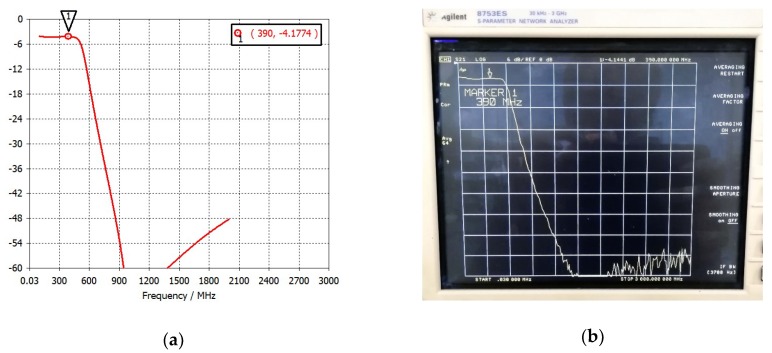
The low-pass filter S_21_ parameter: (**a**) simulation result; (**b**) result measured with the network analyzer.

**Figure 7:**
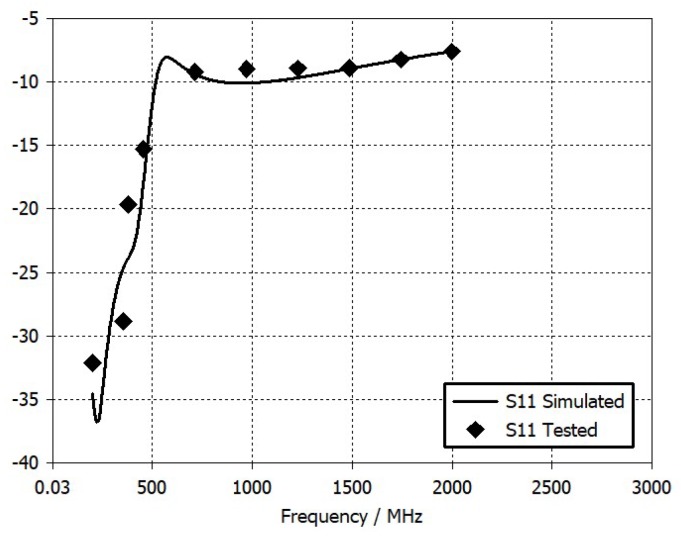
Simulated and tested S_11_ parameters of the low-pass filter.

Figure 6 and Figure 7 report the measured S_21_ and S_11_ parameters, respectively, of the filter compared with the result of the design simulation.

The measured and simulated S_21_ parameters agree very well. A 4.14 dB in-band attenuation has been measured at 390 MHz frequency, which is exactly equal to the attenuation obtained by simulations at the same frequency. Likewise, the measured and simulated S_11_ parameters agree well. At 390 MHz, a −19.7 dB value has been measured on the S_11_ parameter, which is excellent from the impedance-matching point of view. Therefore, the obtained results show that the layout implementation of the low-pass filter has been done correctly and the RF test points have been effective in performing the experimental characterization of a single component, even if already integrated in a wider system like the receiver board.

### 5.2. Measure of the Sensitivity Range, Output Power and IF

In this subsection, the results of the board-level functional tests regarding the sensitivity range, the output power and the IF are reported.

**Figure 8:**
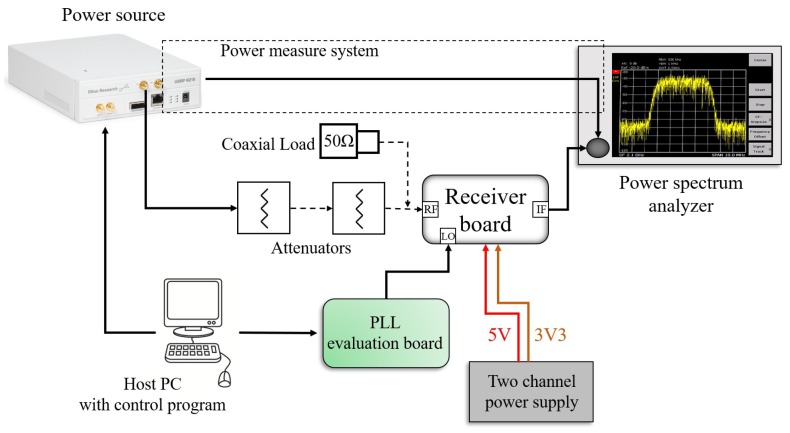
General scheme adopted for the board-level functional tests executed for the RF receiver.

**Figure 9:**
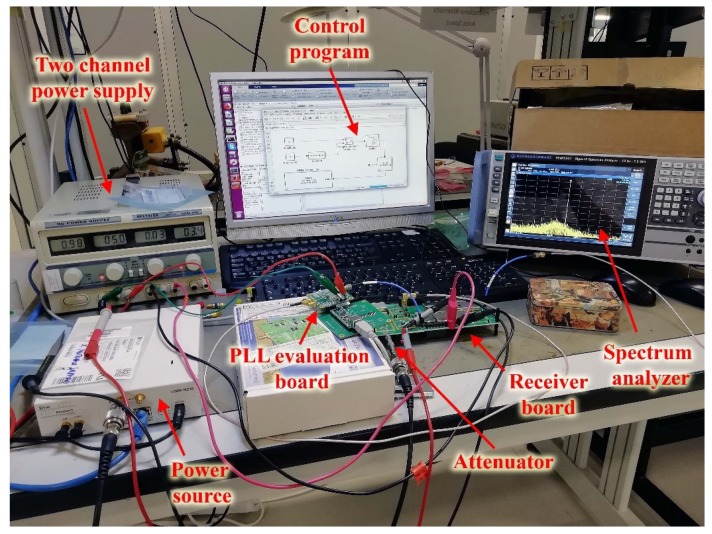
Photograph of the test setup really implemented for the board-level tests executed for the RF receiver.

Figure 8 shows the general scheme of the test setup adopted for the functional tests. Figure 9 shows a photograph of the implemented test setup.

The test setup is constituted by the device under test (the RF receiver), a power source, a spectrum analyzer, a local oscillator, a bench power supply and a host Personal Computer (PC) with a control program.

The receiver test required the generation of a test signal delivered at the RF input port and the reading the 390 MHz output signal at the IF port. The test signal was generated, exploiting a commercial Software-Defined Radio (SDR) as a power source [26], which was configured to generate a Continuous Wave (CW) signal at 2050 MHz frequency, which is within the receiver input frequency range. The output signal was acquired at the IF port by means of a spectrum analyzer [27], which has provided information on frequency, power level, and noise floor.

Regarding the local oscillator, although a COTS Phased-Locked Loop (PLL) was implemented directly on the receiver board, in the first test phase the 1660 MHz LO signal was delivered externally to the mixer LO input using the evaluation board of the same PLL. The signal was brought to the receiver exploiting a debug LO input, which was made through an RF test point. The PLL evaluation board was power-supplied with a unique 5 V voltage and was configured using a dedicated software provided by the manufacturer [28] along with the board. An integer-N configuration was set configuring a 0 dBm power level signal, carefully measured on the spectrum analyzer. The PLL configuration was executed with the same host PC, connected to the evaluation board through a USB A-B cable and an interface board.

The receiver board was power supplied with a 30 V-5 A two-channel bench power supply [29], adjusted to deliver the 5 and 3.3 V voltages. The 5 V voltage was used to supply both the receiver and the PLL evaluation board while the 3.3 V voltage was delivered to the receiver board to power supply some auxiliary functions of the AGC amplifiers. The 5 V and 3.3 V voltages were brought to the receiver by means of dedicated test points directly implemented on the board.

The test was executed in two steps: in the former, the input power level was finely adjusted to generate CW signals from −115 dBm to −70 dBm with a 2.5 dB step; in the latter, the CW signal was applied at the receiver RF input and measured the IF output with the spectrum analyzer. Tuning of the input power level was obtained by combining the SDR gain control (performed by the control program) with the use of one or two 30 or 60 dB external coaxial attenuators. The correct tuning was carefully checked, connecting the SDR directly on the spectrum analyzer input through a coaxial cable and measuring the power level. For each input power level, the IF output signal was measured.

**Figure 10:**
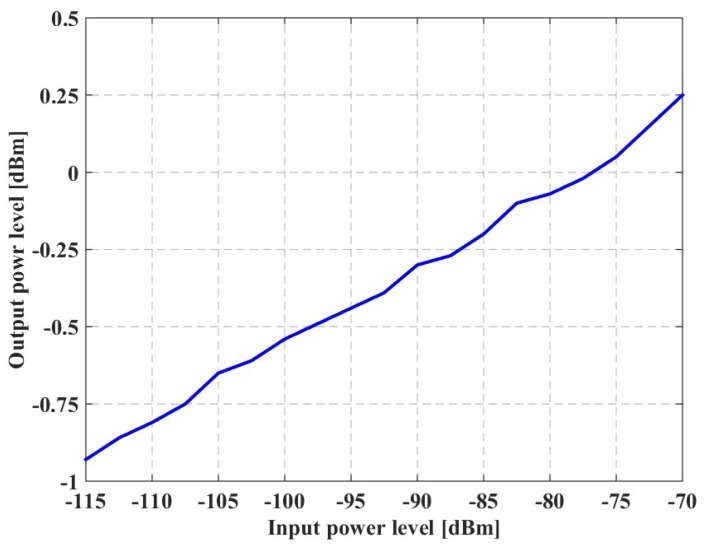
Measured input dynamic range of the receiver board for from −115 dBm to −70 dBm with a 2.5 dB step.

Figure 10 shows a graph that reports the output power level (vertical axis) as a function of the input power level (horizontal axis).

The graph shows that, for input power levels from −115 to −70 dBm, the output power level is within the −1–+1 dBm range. Moreover, for each input power level delivered at 2050 MHz, the IF output signal appeared at 390 MHz frequency on the spectrum analyzer.

These results prove that the system-level requirements regarding the sensitivity range, output power, and the value of the IF have been fulfilled.

### 5.3. Measure of the DC Power Absorption

In this subsection, the measure of the receiver DC power absorption is reported. The test setup is the same as shown in Figure 9. As shown in the figure, the absorbed current is equal to 0.98 A on the 5 V output channel, and 30 mA on the 3.3 V one. Therefore, the total DC power absorption is about 5 W. However, the absorbed power also considers the presence of the PLL evaluation board, which is implemented inside a linear voltage regulator to generate the 3.3 V voltage, starting from the 5 V input voltage. This 3.3 V voltage is not used to power supply the receiver, and is only used for the PLL normal operation and other minor components. Therefore, the current absorption, due to this 3.3 V voltage (which has been estimated equal to 84 mA reading the datasheet), must not be considered in the total DC power absorption of the receiver.

The DC power dissipated (P_D_) by the voltage regulator was estimated as equal to 142.8 mW using Equation (1)

PD ≅ (VIN − VOUT) * IOUT,(1)

where V_IN_ is the regulator input voltage, and V_OUT_ and I_OUT_ are the regulator output voltage and current, respectively. Therefore, the real DC power absorbed by the receiver has been calculated by subtracting to 5 W the power dissipated by the PLL evaluation board (142.8 mW), obtaining a total DC power absorption of about 4.86 W. This value is higher than the system requirement (3.5 W).

### 5.4. Measure of the Noise Figure

The Noise Figure (NF) measurement was performed by adopting the same scheme depicted in Figure 8. In this case, a 50 Ω coaxial load was applied at the 2050 MHz RF input connector. The receiver board was normally power supplied and the external LO signal was delivered through the PLL evaluation board.

**Figure 11:**
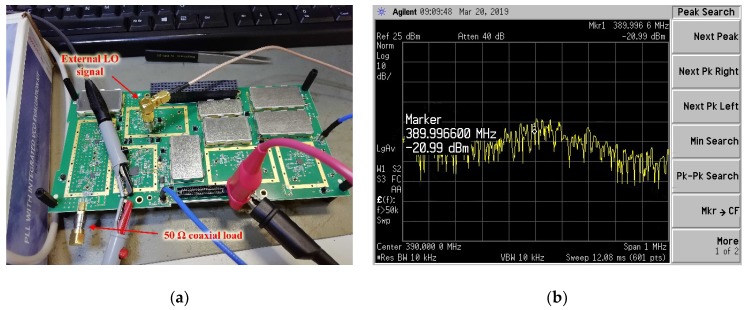
Photograph of the noise figure test setup (**a**) and output noise power screenshot at 390 MHz of the receiver board (**b**) with the RF input terminated with a 50 Ω coaxial load.

Figure 11a shows a photograph of the test setup. Figure 11b shows a screenshot of the noise power measured with the spectrum analyzer [30]. A marker was placed at the 390 MHz central frequency.

Starting from the noise power evaluation, the noise figure has been calculated using Equation (2),

NF = NOUT − 10log_10_(RBW) + 174 − GS, (2)

where N_OUT_ is the noise power, RBW is the instrument resolution bandwidth, and G_S_ is the system gain. The system gain has been calculated, starting from the theoretical gain obtained by system simulations (i.e., 112.4 dB when the input power level is −115 dBm). The total loss of the 50 Ω impedance lines analyzed in [17] and of the RF test points has been subtracted from the gain value. Assuming a 0.05 dB loss for each RF test point [24], and as there are 20 RF test points on the signal path, the system gain has been assumed to be equal to 112.4 − 0.73 − (20 × 0.05), i.e., 110.67 dB. Using Figure 11b and Equation (2), the receiver noise figure has been estimated to be equal to 2.34 dB, which is smaller than the system requirement (3 dB).

## 6. Discussion

The results obtained by the experimental tests have highlighted both the effectiveness of the modeling methodology reported in [17] and the ability to make the board suitable for the characterization of single components. The compliance of the experimental results with the requirements is summarized in Table 1.

Except for the DC power absorption, all high-level requirements have been satisfied. The DC power absorption requirement has not been fulfilled because the receiver was designed without considering the reception system of the signal at the intermediate frequency, which digitalizes it by an ADC. In other words, the output power requirement is set a priori without considering the ADC noise performance, although even the ADC can be characterized in terms of noise figure, like the other RF components [2]. If its noise model is included into the receiver system model, the amplification net gain of the receiver could decrease. A lower amplification gain would reduce the number of the active elements and, therefore, satisfy the DC power absorption requirement.

Regarding the component-level tests, the adopted methodology is the most innovative aspect, especially if it is adopted for the radiation tests. When the COTS components are used, the radiation tests are foreseen in the final phases of the project, when all boards of the whole system are assembled. In this phase of the project, a fault in the system caused by the ionizing radiations would prevent the single component that has generated the failure from being found. A typical approach to discover the radiation behavior of the components is to test them using test boards (or the same evaluation boards) and to characterize the components with functional tests. The parameters obtained by functional tests after radiation help to improve the accuracy of the system model. Such an approach has a high cost because the radiation tests should be repeated for each element used to design the system.

Instead, proving that the RF test points can be used to test a single component (as shown in the example of the filter characterization presented in this paper), even if it is already assembled on the board, shows that the test approach can be used to characterize individual components after the exposition to radiation. The great advantage of this is the fact that the board can be irradiated only once, whilst still maintaining the possibility of discovering the behavior of all components individually, which is very attractive for the reduction in costs. Therefore, by means of the RF test points, the RF components can be checked in order to perform debug operations and to find the component that has caused the fault after a radiation test. Moreover, RF components could be characterized without purchasing evaluation boards, which can be 10 times more expensive than the component itself and are not always available on the market.

Finally, to complete the test methodology, a system of mechanical switches has been also foreseen on the board to allow to switch on and off only components under test. In addition, the tests are possible even in the presence of the shields, since they have been selected with a removable cover.

## 7. Conclusions

In this paper, the experimental results of the receiver prototype designed for Cubesat applications have been reported. The tests have been executed both at a component-level and at board-level. Regarding the component-level tests, an example of the characterization of a custom low-pass filter has been reported. The characterization has been performed measuring the S_21_ parameter with a network analyzer connected to the input and output port of the component by means of the RF test points. The measured S_21_ parameter agrees very well with the simulated one, exhibiting a 4.14 dB in-band attenuation at 390 MHz frequency, which is exactly equal to the attenuation obtained by simulations. Such a result has shown that the design approach allows to test the components individually after they are mounted on the board, which could be used to perform radiation tests and to discover the behavior of the COTS RF components for debugging purposes. Performing the component-level radiation tests through a unique board-level radiation test is extremely attractive to reduce design costs and avoid the purchase of expensive evaluation boards.

Regarding the board-level tests, compliance with high-level requirements has been successfully verified. A CW signal with a power level within −0.93–+0.25 dBm has been measured at the IF output port at a 390 MHz frequency for a sensitivity range of −115–−70 dBm. Moreover, a 2.34 dB noise figure and a 4.86 W DC power absorption have been measured.

## Figures and Tables

**Table 1:** Compliance of the receiver test results with the high-level requirements.

Requirement	Test Result
Sensitivity range: −115–−70 dBm	P_IN_ = −115 dBm → P_OUT_ = −0.93 dBm P_IN_ = −70 dBm → P_OUT_ = +0.25 dBm
Noise figure: <3 dB @ −115 dBm	NF = 2.34 dB
IF: 390 MHz	IF = 390 MHz
IF output power: 0 dBm ± 1 dB	−0.93 dBm ≤ P_OUT_ ≤ +0.25 dBm
DC power absorption: <3.5 W	P_A_ = 4.86 W
